# Truth: explanation, success, and coincidence

**DOI:** 10.1007/s11098-017-0909-2

**Published:** 2017-05-03

**Authors:** Will Gamester

**Affiliations:** School of Philosophy, Religion and History of Science, University of Leeds, Woodhouse Lane, Leeds, LS2 9JT England, UK

**Keywords:** Truth, Deflationism, Inflationism, Success, Explanation, Coincidence

## Abstract

Inflationists have argued that truth is a causal-explanatory property on the grounds that true belief facilitates practical success: we must postulate truth to explain the practical success of certain actions performed by rational agents. Deflationists, however, have a seductive response. Rather than deny that true belief facilitates practical success, the deflationist maintains that the sole role for truth here is as a device for generalisation. In particular, each individual instance of practical success can be explained only by reference to a relevant instance of a T-schema; the role of truth is just to generalise over these individualised explanations. I present a critical problem for this strategy. Analogues of the deflationist’s individualised explanations can be produced by way of explanation of coincidental instances of practical success where the agent merely has the right false beliefs. By deflationary lights, there is no substantive explanatory difference between such coincidental and non-coincidental instances of practical success. But the non-/coincidental distinction just is an explanatory distinction. The deflationist’s individualised explanations of non-coincidental instances of practical success must therefore be inadequate. However, I argue that the deflationist’s prospects for establishing an explanatory contrast between these cases by supplementing her individualised explanations are, at best, bleak. The inflationist, by contrast, is entitled to the obvious further explanatory premise needed to make sense of the distinction. As such, pending some future deflationary rejoinder, the deflationary construal of the principle that true belief facilitates practical success must be rejected; and with it the deflationary conception of truth.

## Introduction

How does a semantic property like truth fit into a world that is fundamentally non-semantic? The deflationary response to this challenge is to deny that there is really a challenge here at all. The nature of truth, we are told, is in some way exhausted by all the instances of a schema such as the disquotational schema (DS) or equivalence schema (ES)^1^:

(DS): ‘p’ is true iff p.
(ES): <p> is true iff p.

Why should we accept the deflationist’s proposal? After all, we wouldn’t accept such schematic, listiform analyses of just any non-fundamental property (for illustration, see the discussion of valence in Field (1972: 362–363)). The difference, says the deflationist, is that truth is not an explanatorily potent property: we do not need to postulate it in order to explain any phenomena. Instead, truth—or, more precisely, the *expression* ‘is true’—is merely an “expressive” linguistic device, useful for indirect reference to, and compendious generalisation over, sentences or propositions. ‘True’ is thus more akin to a logical expression like ‘and’ than a normal predicate like ‘valence’.

The Success Argument seeks to refute this anti-explanatory contention on the grounds that true belief facilitates practical success: we thus need to postulate truth in order to *explain* the practical success of certain actions performed by rational agents (§2). The deflationist, however, has a seductive response (§§3–4). Each individual instance of practical success, we are told, can be explained without reference to truth itself, but only to the relevant instance of the deflationist’s preferred schema; truth then merely generalises over these individualised explanations. I present a critical problem for this strategy (§§5–12). Analogues of the deflationist’s individualised explanations apply as much to *coincidental* instances of practical success where the agent merely has the right false beliefs as they do non-coincidental instances where the agent has true beliefs (§§5, 7). By deflationary lights, then, there is no substantial explanatory difference between such coincidental and non-coincidental instances of success. But the non-/coincidental distinction *just is* an explanatory distinction (§6). The deflationist’s individualised explanations of non-coincidental instances of practical success must therefore be inadequate. However, I argue that the deflationist’s prospects for establishing an explanatory contrast between these cases by supplementing her individualised explanations are, at best, bleak (§§8–11). (Indeed, I argue that *any* truth-free explanation of practical success will struggle to make sense of the *correlation* between true belief and practical success (§12).) The inflationist, by contrast, is entitled to the obvious further explanatory premise needed to make sense of the distinction. As such, pending some future deflationary rejoinder, the deflationary construal of the principle that true belief facilitates practical success must be rejected; and with it the deflationary conception of truth.

## The Success Argument

The Success Argument for inflationism maintains that we must postulate truth to explain the practical success of certain actions performed by rational agents. Let’s illustrate this via the easiest gameshow in the world: *Be a Millionaire!* The contestant sits with two opaque boxes in front of her—box X and box Y. The host puts a cheque for a million pounds into one of the boxes. The rules dictate that all the contestant has to do is pick the box with the cheque in, and she wins the money. Both Alison and Bryan—desperate to be millionaires—go on the gameshow, but have quite different experiences:

**Alison**. The host puts the cheque in box X. Alison chooses box X. She wins the money.

**Bryan**. The host puts the cheque in box X. Unbeknownst to Bryan, the cheque is surreptitiously moved to box Y. Bryan chooses box X. He doesn’t win the money.

The actions share a distinctive feature, which we will call *psychological pertinence* 2:

*Psychological pertinence*

An action A is psychologically pertinent to an outcome O for a rational agent S in a context C iff: (i) A is an option for action for S in C; and (ii) S believes that S’s A-ing in C will result in O.

The basic idea is simple: for any agent in any context, the action psychologically pertinent to a particular outcome is the one that the agent believes will result in that outcome if she were to perform it. This means that, if the agent desires that O and has no countervailing desires, then the agent will perform the action psychologically pertinent to O. We can say that an action A is psychologically pertinent with respect to a desire D (for S in C) iff D is the desire that O and A is psychologically pertinent to O (for S in C). Psychological pertinence is thus a necessary condition on intentional explanation of rational action: if we cite a set of beliefs and a desire in the intentional explanation of an agent’s action, then (on the assumption that the agent acted rationally) we are claiming that the action was psychologically pertinent with respect to the desire according to those beliefs.^3^

The slightly clunky locution ‘S’s A-ing in C will result in O’ is left deliberately vague in order to cover the myriad different connections that an agent might imagine holds between her performing an action and an outcome coming about (via magic or the will of God, for example, as well as more humdrum causal connections, or even a giant question mark if one is unsure of why the one will bring about the other). Whatever else she believes, the agent must believe that her performance of A will be in *some* way explanatorily relevant to O coming about. For this reason also, the ‘will result in’ locution is preferable to simple conditional ‘if S As in C then O’.

Because their relevant beliefs are effectively identical (we can stipulate) and both have the desire for wealth, the same action is psychologically pertinent with respect to the relevant desire for both Alison and Bryan. However, only Alison’s action actually satisfies her desire. Thus Alison’s action has a feature that Bryan’s lacks, which we will call ‘effective pertinence’:

*Effective pertinence*

An action A is effectively pertinent to an outcome O for an agent S in context C iff S’s A-ing in C will result in O.

That is, an action is effectively pertinent to an outcome if performing that action will *in fact* result in that outcome. An action A is therefore effectively pertinent with respect to S’s desire D (in C) iff D is the desire that O and A is effectively pertinent to O (for S in C).

Note that, for an action to be effectively pertinent with respect to an agent’s desire, it need not be the case that the agent performs the action at all—and even if they do, it is not necessary that they perform the action *because* they have that desire. For example, drinking this coffee will mean that I am more alert for the upcoming seminar (let’s say), which I certainly want to be. The action itself is therefore effectively pertinent with respect to this desire even if I (i) don’t drink the coffee; (ii) drink the coffee despite being unaware of its caffeine-based benefits, because I like the taste (for example); or (iii) have the relevant belief about these benefits, but nonetheless drink the coffee for a different reason, e.g. whilst under duress.

Nonetheless, with effective pertinence in hand it is tempting to introduce a definition of *success*, understood as a species of effective pertinence:

*Success*

An action A is successful with respect to a desire D for an agent S in a context C iff: (i) S’s A-ing in C is effectively pertinent with respect to D; and (ii) D correctly features in the intentional explanation of S’s A-ing in C.

The idea is just that an action is successful iff it satisfies an agent’s desire, and the agent performed that action *in order to* satisfy that desire.^4^ Note that actions are successful *with respect to desires*; this is because it seems implausible that it will always be the case that exactly one desire correctly features in the intentional explanation of an action: beer drinkers will often insist that they drink beer not only because they want to get drunk, but *also* because they like the taste. Furthermore, it’s entirely possible that such an action will be effectively pertinent with respect to one of these desires and not the other, e.g. if the beer tastes nice, but is secretly non-alcoholic (or, more usually, is gross but gets you drunk). Perhaps an action can be said to be successful *simpliciter* iff it is successful with respect to every desire that correctly features in its intentional explanation—we have no direct need for such a notion.

Given that any action performed by a rational agent will be psychologically pertinent with respect to the desire(s) that correctly feature in its explanation, if an action A is successful with respect to a desire D (for S in C) then the psychological and effective pertinence of A with respect to D coincide (for S in C).^5^ Nothing hangs on this explication of ‘success’ being the “correct analysis” of this concept, if such there be—if one has quibbles about cases, that should not defeat our purpose. All that the argument strictly requires is this coincidence of psychological with effective pertinence of an action with respect to a desire that features in the explanation of that action: the agent performing an action because she thinks it will result in a particular outcome, and the action actually resulting in that very outcome.

Alison’s action is not only psychologically pertinent with respect to her desire for wealth, but also effectively pertinent with respect to this desire; indeed, it is *successful* with respect to this desire. Bryan’s is not. And this despite the fact that both agents have equivalent, equally well-justified beliefs with respect to their present situation, and thus which action would result in the satisfaction of their desire for wealth. The sole difference between the cases is that Alison’s belief about the location of the cheque was true while Bryan’s was false. Thus the inflationist about truth *wants* to say that truth plays a role in explaining why Alison’s action turned out to be effectively pertinent: Alison’s action turned out to be successful *because Alison’s beliefs were true*. In particular, the beliefs that feature in the intentional explanation of the action—those that explain the psychological pertinence of the action—were true.

The onus is on the deflationist at this point. Actions that result from true beliefs (via psychological pertinence) tend to be effectively pertinent with respect to the desires that feature in their intentional explanations along with these beliefs—that is, they tend to be successful. This seems like a prime explanatory role for truth. The deflationist thus owes us an account of how we can possibly explain the success of Alison’s action *without* appealing to the truth of her beliefs.

## The deflationist's response 1—individualised explanations

The deflationist, however, has a seductive response.^6^ She does not deny that true beliefs facilitate practical success; instead, the strategy is to *earn the right* to this claim on deflationist-friendly grounds, by arguing that the sole role for the truth predicate here is as a device for generalisation. To this end, we are told that, strictly speaking, any mention of ‘truth’ can be eliminated from the explanation of each *individual* instance of practical success in favour of the relevant instance of the deflationist’s preferred schema. The truth predicate merely exploits this structural similarity, and thereby generalises over these individualised explanations.

The deflationist asks us to consider any individual instance of practical success. Horwich’s (1998: 22–23) example is of an agent, Bill, who desires a beer and believes he can get one simply by nodding—which, in fact, he can. The deflationist’s explanation of Bill’s success is as follows:

**Table:** 

(1) Bill desires that Bill has a beer	*premise*
(2) Bill believes that Bill’s nodding will result in Bill having a beer	*premise*
(3) For any rational agent S and option for action A, if S believes that S’s A-ing will result in O and S desires that O, then (other things being equal) S will A. (For simplicity I suppress relativising to a context)	*Psychological Law*
(4) Bill is a rational agent	*premise*
(5) If Bill believes that Bill’s nodding will result in Bill having a beer and Bill desires that Bill has a beer, then (other things being equal) Bill will nod	*from (3), (4)*
(6) Bill nods	*from (1), (2), (5)*
(7) Bill’s nodding will result in Bill having a beer	*premise*
(8) Bill has a beer	*from (6), (7)*
(9) Bill’s desire that Bill has a beer is satisfied	*from (1), (8), definition of satisfaction*

I have tried to make the deflationist’s argument as explicit as possible. (1) and (2) are treated as premises, although they themselves could in principle be explained, presumably via Bill’s psychological history. The content of the belief in (2) is usually given in conditional form:

(2*): Bill believes that: if Bill nods, then Bill has a beer.

The same goes for (7). Once again, the ‘will result in’ locution allows us to make explicit that Bill must believe that there’s an explanatory connection between his nodding and his obtaining a beer; and, in (7), there must actually *be* such a connection.

We are normally presented with just (5), rather than (3), (4), and (5). However, for (5) to truly be explanatory it cannot simply be a *sui generis* fact about Bill that when he has that belief and desire he will perform that action. Rather, it must be an instance of a more general psychological phenomenon (and it is clear that this is how Horwich intends for it to be understood). This is given by the “practical syllogism” of (3), along with the assumption that Bill is a rational agent (4). I have also made explicit the *ceteris paribus* clause. Note that (3) essentially tells us that, if an action is psychologically pertinent with respect to an agent’s desire (for that agent in that context), then other things being equal she will perform that action.

Given the *ceteris paribus* clause, the inference from (1), (2), and (5) to (6) is not deductive, but as we are treating other things as being equal, we can simplify things by treating it as such.

(7) is also a premise of the case, but it’s important to emphasise that (7), like (5), cannot be explanatory if it is somehow a *sui generis* fact about Bill’s nodding—it must itself be explicable. Unlike in the case of (5), there is no single obvious candidate for this explanation, and the right explanation will depend entirely on how we flesh out the details of the case. For instance, it might be that Bill is in a bar where the bartender is aware of Bill’s introverted nature and drink preference, and thus will give Bill a beer when he nods; or perhaps Bill has attached his head to a Wallace-and-Gromit-style beer-dispensing device, such that when he nods he gets a beer. The details do not matter; but that there *are* such details does.

The rest of the inference requires no further elaboration.

## The deflationist's response 2—generalisation

The inference given in the argument from (1) to (9) is the lynchpin of the deflationist’s response to the Success Argument. The deflationist hereby provides what we might call a “two-step” account of Bill’s success. We start with *an explanation of why Bill performed a certain action* in (1) through (6). This explanation goes via the psychological pertinence of the action—given in (2)—with respect to a desired outcome (1). Since we know that Bill, qua rational agent, will (other things being equal) perform actions psychologically pertinent to his desires (as stated in (5), obtained via (3) and (4)), Bill’s propositional attitudes hereby explain why Bill nodded (6). The second step involves *an explanation of why this action ended up satisfying Bill’s desire* in (6) through (9). This explanation goes via the effective pertinence—given in (7)—of the action (6) to the desired outcome (8). *Together*, the two steps are supposed to provide a full explanation of the *success* of Bill’s action. Bill performed the action because it was psychologically pertinent to an outcome he desired, and the action resulted in the satisfaction of this desire because it was, in fact, effectively pertinent to this outcome.

The critical premises are (2) and (7):

(2): Bill believes that Bill’s nodding will result in Bill having a beer.
(7): Bill’s nodding will result in Bill having a beer.

There are at least three important features to note.

First, (2) effectively states the *psychological pertinence* of Bill’s nodding with respect to his desire for a beer (for Bill in the present context); while (7) states the *effective pertinence* of this action with respect to this desire. Second—as noted above—while (2) and (7) are included as premises, they can in principle be explained themselves; that is, we can *explain* the psychological and effective pertinence of the action. This will be important later.

Most significant in the present context is how (2) and (7) relate to the deflationist’s conception of *truth*. Recall that the deflationist maintains that the nature of truth is somehow exhausted by all the instances of a schema such as (DS) or (ES), which take sentences and propositions as the primary truth bearers, respectively. To say of any *particular* sentence or proposition that it is true is just to say what appears on the RHS of the schema. The truth of ‘snow is white’ or <snow is white> is exhausted by snow’s being white. The truth of a *belief* is then understood derivatively from this: to say of any particular *belief* that it is true is just to say that it is the belief that p *and* (in fact) p.^7^ In particular, to say of Bill’s belief that it is true is just to say that Bill believes that Bill’s nodding will result in Bill having a beer, and (in fact) Bill’s nodding will result in Bill having a beer. The central observation is that this conjunction *is just the conjunction of (2) and (7)*.

So, by the deflationist’s lights, the “truth” of Bill’s belief *does* make an appearance in the explanation of Bill’s success; but importantly this does not require anything more than is given by the deflationary schema. By the very nature of the schema, the truth of an “action-guiding” belief like (2) will *consist in* the psychological and effective pertinence of the relevant action with respect to the relevant desire.^8^ And this is what gives the deflationist’s purported explanation its seductive charm, for given the psychological and effective pertinence of the action, then as long as the agent is rational, the resultant action will be successful. Furthermore, it seems as though this style of explanation is entirely generalizable: after all, for any successful rational action there will be a psychological explanation of why the agent performed that action and a “worldly” explanation of why it resulted in the satisfaction of the desire. Therefore, the deflationist argues, when we say that true belief facilitates practical success, the role of the truth predicate is simply one of generalisation: to generalise over these particularised explanations.

The deflationist’s response has not been without its critics; but the argument is incredibly seductive precisely because it is difficult to see what could be *missing* from the deflationist’s (1)-(9) explanation of Bill’s success. There simply seems to be no explanatory work left for an inflationary truth property to do; so it becomes very tempting to think that truth must merely be generalising over these particularised explanations.^9^ My goal in the rest of this paper is to argue directly that these individualised explanation are, in fact, problematically incomplete.

## A problem case

Let us introduce our problem case; a third contestant, Callum:

**Callum**. The host puts the cheque in box X. Unbeknownst to Callum, the cheque is surreptitiously moved to box Y. Callum chooses box X. In the audience is an eccentric billionaire, who loves box X. She is so pleased that Callum has chosen her favourite box that she gifts him a million pounds.

Callum is like both Alison and Bryan in that his action is psychologically pertinent with respect to his desire for wealth. He is like Bryan, but *unlike* Alison, in that his belief about the location of the cheque (which explains the psychological pertinence) is false. But he is like Alison, and *unlike* Bryan, in that his action *is* effectively pertinent—indeed, *successful*—with respect to his desire for wealth.

Despite this alignment of psychological and effective pertinence, there is a salient difference between Callum’s success and Alison’s success. While Alison’s success is to be expected, Callum’s is quite out of the blue—the fact that the action he *thought* would result in the satisfaction of his desire *in fact* turned out to do so is a *sheer coincidence*.

To be clear, there is nothing coincidental or inexplicable about *the fact that Callum performed a certain action*; nor is there anything coincidental or inexplicable about *the fact that Callum’s action had the effects it had*, in and of themselves. Each of these can be explained. It is Callum’s *success* that is coincidental, i.e. that *the same* action turned out to be *both* psychologically and effectively pertinent for Callum. Alison’s success, by contrast, is a paradigmatic non-coincidence.

## Coincidence

There is nothing particularly special about the existence of coincidental instances of practical success in and of themselves. The non-/coincidental contrast is ubiquitous. It is probably a coincidence that the Sun and the Moon appear to be roughly the same size from the Earth; though it might be the result of intentional design. Two people may look alike by sheer coincidence; or they might be relatives. I may have pressed my hands into some wet concrete and thereby left an imprint into which my hands perfectly fit; or the rain may have eroded that shape into the concrete by chance, making the perfect fit sheer coincidence.

The significance of the contrast for our purposes is that it is often treated as a truism that coincidences *cannot be explained*.^10^ In fact, my argument only requires the weaker, and necessarily more plausible, claim that there is a difference in kind between what *can* be said by way of explanation when something is a coincidence, and when it is a non-coincidence. For of course there is *something* we can say by way of explanation when something is a coincidence. If it is a coincidence that the Sun and the Moon appear to be the same size, then the best we can do is offer an explanation of why the Sun appears to be that size (in terms of its size and distance from Earth) and a distinct explanation of why the Moon appears to be that size (in terms of *its* size and distance from Earth), and conjoin them together. That is, the best we can do is conjoin two *distinct* explanations of each of the relevant incidences that make up the *co*incidence. This quasi-explanatory pattern is pervasive. *If it is a coincidence* that two people look alike, all we can offer is an explanation of why one person looks the way they do (in terms of genes and environment) conjoined with an explanation of why the other person looks the way they do (in terms of genes and environment). *If it is only a coincidence* that there is an imprint in the concrete that my hands fit perfectly into, then all we can offer is an explanation for why my hands are a certain shape (in terms of genes and environment) and an explanation of why the imprint is a certain shape (in terms of erosion). Since all it is for there to be a perfect fit between two things is for one to have one shape and the other to have another, the conjunction of these distinct explanations is all that can be said by way of explanation, *if the fit is merely coincidental*.

We can call these *conjunctive quasi*-*explanations*. In line with the aforementioned truism, it does not seem especially plausible to me that such conjunctive quasi-explanations really deserve to be called ‘explanations’, so perhaps ‘pseudo-explanations’ would be better; but we’ll use ‘quasi’ in the interests of neutrality.^11^ All that matters for our purposes is that there is an obvious difference in kind between such conjunctive quasi-explanations (be they explanations or not) and explanation proper^12^; so the kind of conjunctive quasi-explanation that may be tolerable qua explanation when something is a coincidence is manifestly inadequate when it is not a coincidence. For example, if asked why two people look alike we *merely* explain why each looks the way they do, and leave out the fact that they are related, then we have portrayed what is perfectly explicable as a coincidence, and thereby failed to explain why it is they look alike. Similarly if we explain why each of the Sun and Moon appear to be the size they do in terms of their size and distance from Earth, without mentioning the intentions of a creator. To offer a conjunctive quasi-explanation of a non-coincidence is a straightforward explanatory failing. In particular, until the quasi-explanation is *supplemented* with something that *actually explains* the non-coincidence, it remains unexplained, precisely because we hereby portray what is perfectly explicable as though it is coincidental.

Bearing this explanatory contrast in mind, my contention is that the deflationist’s refusal to concede the explanatory potency of truth means that she does not have the resources to distinguish the non-coincidental instances of practical success from the coincidental.

## The problem

Recall Alison, our successful gameshow contestant. By the deflationist’s lights we can give a complete two-step explanation of Alison’s success via the following derivation:

**Table:** 

(1A) Alison desires that Alison has a million pounds	*premise*
(2A) Alison believes that Alison’s choosing box X will result in Alison having a million pounds	*premise*
(3A) For any rational agent S and option for action A, if S believes that S’s A-ing will result in O and S desires that O, then (other things being equal) S will A	*Psychological Law*
(4A) Alison is a rational agent	*premise*
(5A) If Alison believes that Alison’s choosing box X will result in Alison having a million pounds and Alison desires that Alison has a million pounds, then (other things being equal) Alison will choose box X	*from (3A), (4A)*
(6A) Alison chooses box X	*from (1A), (2A), (5A)*
(7A) Alison’s choosing box X will result in Alison having a million pounds	*premise*
(8A) Alison has a million pounds	*from (6A), (7A)*
(9A) Alison’s desire that Alison has a million pounds is satisfied	*from (1A), (8A), definition of satisfaction*

(1A)–(6A) explains why Alison performed a particular action, and (6A)–(9A) explains why that action resulted in the satisfaction of her desire. In Bryan’s case, we can provide the first step of the explanation, but not the second.

The *problem* is that we can apply the deflationist’s explanatory schema, without alteration, to Callum’s case:

**Table:** 

(1C) Callum desires that Callum has a million pounds	*premise*
(2C) Callum believes that Callum’s choosing box X will result in Callum having a million pounds	*premise*
(3C) For any rational agent S and option for action A, if S believes that S’s A-ing will result in O and S desires that O, then (other things being equal) S will A	*Psychological Law*
(4C) Callum is a rational agent	*premise*
(5C) If Callum believes that Callum’s choosing box X will result in Callum having a million pounds and Callum desires that Callum has a million pounds, then (other things being equal) Callum will choose box X	*from (3C), (4C)*
(6C) Callum chooses box X	*from (1C), (2C), (5C)*
(7C) Callum’s choosing box X will result in Callum having a million pounds	*premise*
(8C) Callum has a million pounds	*from (6C), (7C)*
(9C) Callum’s desire that Callum has a million pounds is satisfied	*from (1C), (8C), definition of satisfaction*

It is striking that (1C)–(9C) may well be an acceptable conjunctive quasi-explanation of Callum’s success. Its familiar two-step form explains why he performed a certain action, and why that action resulted in the satisfaction of his desire. This is presumably all there is to say by way of explanation *when someone’s success is coincidental*.

But Alison’s success is not coincidental. As such, there must be some explanatory contrast between Alison and Callum; something that *explains why* Alison’s action was successful, but which cannot explain why Callum’s action was so. However, if the deflationist is right that *all* there is to the explanation of Alison’s success is (1A)–(9A), just as *all* there is to the explanation of Callum’s success is (1C)–(9C), then there is no such contrast: the two are on an explanatory footing. If this is right, then we lose all right to say that there is any non-/coincidental contrast between the cases; and this is a straightforwardly disastrous result. To treat every instance of practical success—coincidental and non-coincidental—in this explanatorily identical way is to treat the entire phenomenon as coincidental and hence mysterious. What this shows is that we must go *beyond* the (1)–(9) schema if we are to truly *explain* the non-coincidental instances of practical success, and thereby recover the non-/coincidental contrast.

Furthermore, it is simply obvious what the *inflationist* will say at this point. The inflationist will appeal to some explanatory premise that invokes truth: that actions that result from true beliefs tend to be successful (i.e. effectively pertinent with regards to the desires that they result from), for example. Since the beliefs that Alison’s action resulted from are true, this *explains* the success of Alison’s action^13^; and since the beliefs that Callum’s action resulted from are *not* true, it cannot do so for Callum. This is exactly the kind of explanatory tie-breaker we need. Moreover, it’s intuitively very appealing: it simply seems obvious that what explains Alison’s success is that she has true beliefs; while Callum is successful despite having false beliefs. This is why Callum’s success is merely coincidental where Alison’s is straightforwardly explicable.

This move, however, is not open to the deflationist; for the deflationist’s goal is precisely to *deny* this kind of explanatory role for truth. The deflationist is thus stuck with the unenviable task of trying to explain Alison’s success *in particular*, by reference to something that is true of Alison but not true of Callum *other than* the truth of her beliefs. There are broadly two strategies here, both of which we shall consider: either trying to add an additional explanatory premise, as the inflationist does, but which does not appeal to truth; or by extending the schema by looking to the explanations of the explanatory premises used.

But note that, *whatever* the deflationist goes on to say at this point, this is already a major dialectical victory for the inflationist. The deflationist’s goal was to show that truth is merely a device for generalisation in the principle that true belief facilitates practical success by showing that each individual instance of practical success can be explained without reference to truth. The argument was seductive precisely because the (1)–(9) “explanations” seemed to leave no explanatory work for an inflationary truth property to do. But we’ve now seen that the (1)–(9) “explanations” *must* be incomplete, for otherwise we risk collapsing the distinction between coincidental and non-coincidental instances of practical success. And we’ve also seen that the inflationist is entitled to the obvious additional explanatory resources required to recover this contrast. (It is difficult to see what, other than antecedent deflationary commitments, could lead us to resist it.) As far as the deflationist is concerned, then, we are back to square one: she now has to show that we can explain any *non*-*coincidental* instance of practical success without reference to truth; and this is just a slightly finessed version of the original challenge set out by the Success Argument. Until this is done, she cannot maintain that the sole role for the truth predicate is as a device for generalisation.

## Supplementing the explanation? 1

Perhaps the obvious response for the deflationist here is to extend the (1)-(9) schema by explaining some of the explanatory premises used therein, with the goal of recovering the non-/coincidental contrast in this further explanation.

In the non-coincidental case, Alison’s, we have the explanatory premise (2A), which states the psychological pertinence of Alison’s action:

(2A): Alison believes that Alison choosing box X will result in Alison having a million pounds.

That Alison has this belief will be explained using premises like the following:

(2Ai): Alison believes that Alison choosing the box with the cheque in will result in Alison having a million pounds.
(2Aii): Alison believes that the box with the cheque in is box X.

Along with some general principle about how sensible agents like Alison tend to reason given simple premises like these. Meanwhile, our coincidental case, Callum, has the following explanatory premise:

(2C): Callum believes that Callum choosing box X will result in Callum having a million pounds.

That Callum has this belief can equally be explained by analogous premises such as the following:

(2Ci): Callum believes that Callum choosing the box with the cheque in will result in Callum having a million pounds.
(2Cii): Callum believes that the box with the cheque in is box X.

Along with the same general principle. As such, it is important to note that, in each case, *that the agent has the relevant belief* is perfectly explicable; and, given this, it is equally explicable in each case *why the agent performed the relevant action*. The psychological pertinence of each agent’s action is, as we should expect, on an explanatory footing.

Nonetheless, there is an explanatory contrast here. The kind of simple reasoning that agents like Alison and Callum go in for given simple premises like these is valid. In Alison’s case, belief (2Ai) and (2Aii) are both true. Therefore her resultant belief, (2A), is guaranteed to be true. By contrast, Callum’s belief (2Cii) is false. There is no general principle that valid reasoning from false premises will result in a true conclusion; there is thus no guarantee that Callum’s resultant belief, (2C), will be true. And yet it is. *That Callum’s belief is true* is a coincidence.

Assuming that the deflationist is entitled to an adequate notion of validity, she can make sense of this explanatory contrast. In Alison’s case, she can appeal to the general principle that conclusions drawn by valid inference from true premises will be true to *explain* the truth of Alison’s success. There is no such explanation of the truth of Callum’s belief.

Unfortunately, as far as the present argument is concerned, this explanatory contrast is of no use *to the deflationist*. Establishing an explanatory contrast with regards to *the truth of the agents’ beliefs* does not automatically establish an explanatory contrast with regards to *the success of the agents’ actions*. So while the deflationist might, in this way, establish the coincidental status of *the truth of Callum’s belief*, we can then ask what relevance this has to the coincidental status *of Callum’s success*. Here’s one way in which it might be relevant: there may be a general principle that rational actions that result from true beliefs tend to be effectively pertinent with respect to the desires that cause them. We can appeal to this principle to explain the effective pertinence (and hence success) of Alison’s action; not so with Callum. If this is right, then the fact that Callum’s belief (2C) was true by coincidence would clearly be relevant to why his action was successful by coincidence. But this is precisely the explanatory role for truth that the deflationist is denying.

Without such a principle, however, the deflationist cannot hope to attach any explanatory significance to the observation that Callum’s belief (2C) is true by coincidence. There are any number of features that Callum’s belief might have by coincidence: he might have had it at his favourite time of day, or just as someone else formed the exact same thought. Most of these features are not explanatorily relevant to Callum’s success; and, according to the deflationist, neither is the truth of his belief. So while this is obviously where the relevant explanatory contrast lies, the deflationist is not entitled to it, precisely because truth is purportedly *not* explanatory relevant to practical success. Noting this explanatory contrast thus constitutes no progress at all, by deflationary lights.

Moreover, since we can stipulate that Alison and Callum are otherwise identical (they may have identical life histories up to the point that the cheque’s location is switched), there will be no *further* difference in the explanation of the psychological pertinence of each agent’s action; after all, each agent believes that the action will result in the satisfaction of their desire *for exactly the same reasons*. By deflationary lights, then, there is nothing that might explain Alison’s success, and not Callum’s, in the explanation of the psychological pertinence of their actions.

## Supplementing the explanation? 2

The non-coincidental case, Alison’s, also uses premise (7A), which states the effective pertinence of the action:

(7A): Alison choosing box X will result in Alison having a million pounds.

This will be explained by premises such as the following:

(7Ai): Alison choosing the box with the cheque in will result in Alison having a million pounds.
(7Aii): The box with the cheque is in box X.

By contrast the analogous premise in the coincidental case (7C), will permit of an entirely different explanation:

(7C): Callum choosing box X will result in Callum having a million pounds.
(7Ci): Callum choosing the billionaire’s favourite box will result in Callum having a million pounds.
(7Cii): The billionaire’s favourite box is box X.

*That the agent’s action resulted in the agent getting the money* is nonetheless perfectly explicable in both cases, as we should expect.

There is, of course, a radical difference in explanatory detail: one mentions the location of the cheque, the other an eccentric billionaire. But this too is of no use to the deflationist. For mere difference in explanatory detail is insufficient to render one instance a coincidence and the other not. If a successful contestant’s cheque had been placed in box Y instead of box X, then the explanation of the effective pertinence of choosing that box would be *different* (i.e. mentioning box Y rather than box X), but her success would not be coincidental for that. What is it about *those* facts featuring in the explanation of the effective pertinence of Callum’s action is supposed to render his success coincidental? After all, if Callum had *believed* that the eccentric billionaire would act in this way, and had chosen box X on this basis, then his success would have been non-coincidental. So it does not seem to be anything about these facts *in and of themselves* explaining the effective pertinence of the action that makes for an explanatory contrast.

The obvious *structural* difference here is that the beliefs that explain the psychological pertinence of Alison’s action (2Ai) and (2Aii) *match* the worldly facts that explain the effective pertinence of her action (7Ai) and (7Aii)—her action is successful for precisely the reasons she thinks it will be. Not so for Callum. And it is obvious that this is what we *want* to appeal to in accounting for the non-/coincidental contrast between the cases. We *want* to say that an agent’s actions tend to be successful when there is this matching between her beliefs and the world; and so to *explain* Alison’s success with reference to this matching. But this is exactly the inflationist’s contention: that there is a substantive relation that holds between Alison’s beliefs and the world that *explains* her success. The explanatory significance of this matching is *precisely what the deflationist is denying*.

The mere explanation of the effective pertinence of Alison’s and Callum’s actions has thus not turned up anything of explanatory relevance to the practical success of their actions by deflationary lights either. This is unsurprising. For, as emphasised, *that the agent chose box X* is perfectly explicable in each case; as is the fact that *choosing box X made them rich*. What is surprising in Callum’s case is that *the same* action is *both* psychologically and effectively pertinent; and the natural account of this is that there is a relation that holds between the beliefs that explain the psychological pertinence of Alison’s action and the facts that explain its effective pertinence that does not hold in Callum’s case. But the deflationist refuses to explain Alison’s success by reference to this relation.

It is difficult to see how there could be any relevant explanatory contrast in the explanation of any of the *other* premises, besides (2A)/(2C) and (7A)/(7C). I therefore cannot see how the deflationist can hope to recover the non-/coincidental contrast in the explanation of the premises used in the (1)–(9) inferences. At the very least, this leaves the deflationist with all the work to do, while the inflationary move is obvious.

## A clarification

It is worth being clear on the nature of the challenge here. The point is *not* supposed to be that a deflationary truth predicate would be unable to *generalise over* the non-coincidental instances of practical success in particular. The point is just the opposite: the truth generalisation is not *just* a generalisation; it is a *selective* generalisation. In particular, it excludes *coincidental* instances of practical success, like Callum’s, where the agent is merely lucky enough to have the right false beliefs. Instead, it generalises over those *non*-*coincidental* cases of practical success, like Alison’s, where the agent has true beliefs. The question is: what sense can the deflationist make of the non-/coincidental contrast between the cases, given the proposed explanatory redundancy of truth?

The deflationist proposes that the explanatory potency of “truth” is merely the explanatory potency of the psychological fact (S believes that p) and “worldly” fact (p) that makes up the relevant instance of the schema. In particular, there is no explanatorily potent relation that holds between the psychological and worldly fact when they accord with the schema. Accordance with the schema is thus not explanatorily significant, but only expressively significant: it allows the truth predicate to generalise over these cases. The difficulty is that we can *also* provide a conjunction of psychological and worldly facts in coincidental cases like Callum’s; just not ones that accord with the schema. All this seems to entail, by deflationary lights, is that the truth predicate cannot be used to generalise over such cases: by the deflationist’s own lights, this is an *expressive* difference, not an *explanatory* one. If the deflationist maintains that *according with the schema* has some explanatory import that *not according with the schema* does not, then she is conceding the game to the inflationist; for then truth is making an explanatory contribution after all.

Grant, then, that the deflationist can generalise over the non-coincidental instances of practical success; the question is what relevance being able to so generalise has to the non-/coincidental contrast between the cases. Given that this is an explanatory contrast, if the deflationist is right that truth is explanatorily impotent, the answer must be: no relevance whatsoever. Indeed, *this constitutes a problem for the deflationist*, not a solution: why should it be that the cases generalised over using an explanatorily impotent predicate are of a different explanatory status to those excluded by the generalisation?

## Supplementing the explanation? 3

We have seen that the prospects for recovering the non-/coincidental contrast by explaining the premises used in each (1)–(9) inference are bleak. The other option for the deflationist, then, is to add some *further* explanatory premise that explains Alison’s success in particular, but which does not make reference to the truth of her beliefs.

I have said that the inflationist will appeal to an additional explanatory generalisation, along the lines of (EG), to explain Alison’s success:

(EG): Actions that result from true beliefs tend to be successful.

A natural line of response for the deflationist is to try and utilise a schematic thesis where the inflationist seeks to use an explanatory generalisation. In this way, the deflationist may hope to explain the success of Alison’s action on deflationist-friendly grounds. It will be helpful to consider this line of resistance and where it goes wrong. First, it is not clear exactly what schema the deflationist should try and use here. (S1) will not do:

(S1): If an action results from the belief that p and p, then it will tend to be successful.

Not many *single* beliefs can be as useful as (S1) makes out. The deflationist will require something more like:

(S2): If an action results from the belief that p and the belief that q and…, and p and q and…, then it will tend to be successful.

Now, the thought might be as follows. Given some such schema as this, the deflationist can rearticulate it via the deflationary predicate as: ‘Actions that result from true beliefs tend to be successful’; or, in other words, as something very close to precisely the generalisation the inflationist uses as an explanatory tie-breaker. Given this—the reasoning might go—she is as entitled to the non-/coincidental contrast as the inflationist.

However, even if the deflationist can articulate some such principle using some such schema, a moment’s reflection reveals that the deflationist cannot use this as an explanatory tie-breaker, as the inflationist does. For recall the deflationary strategy: to *earn the right* to the claim that true belief facilitates practical success by *first* explaining (or providing a recipe for explaining) each individual instance of practical success; and *then* generalising over them. It is only in this way that she can maintain that the sole role for truth is as a device for generalisation. So *for the deflationist* the generalisation necessarily comes later in the explanatory order; she thus cannot appeal to it in the explanation of individual instances of practical success, as the inflationist does (EG). It is also worth explicitly mentioning that (S2) *itself* cannot be invoked in the explanation of any instance of practical success; for—lest we forget—(S2) is a *schema*. It is only the *instances* of a schema that have content.

To reiterate the point from above, it may well be the case that (S2) (or some schematic thesis like it) has many true instances; that these instances include the relevant non-coincidental instances of practical success; and thus that the deflationary truth predicate can generalise over these instances. As mentioned, however, the mere fact that certain non-coincidental cases accord with a schema does not (by deflationary lights) *explain* why they are non-coincidental; it *merely* allows them to be generalised over. There are certainly coincidental cases, like Callum’s, that do *not* accord with the schema. But the challenge to the deflationist is to make sense of the fact that these cases are of a different explanatory status (being coincidental), without conceding that the schema latches onto an explanatorily potent property.

The deflationist therefore cannot use a schematic thesis like (S2) (or a rearticulation of it formulated via a deflationary truth predicate) to break the explanatory deadlock here. And, by design, there is very little *else* that differs between Alison and Callum, and thus very little else which might be invoked to establish an explanatory contrast between the two. While the deflationist is able to generalise over the non-coincidental cases of practical success and exclude the coincidental cases, she is unable to say what this explanatory contrast *consists in*.

## The inadequacy of mere generalisation

Indeed, we can sharpen the point against the deflationist here. For the deflationist is keen to emphasise the distinction between explanation of a phenomenon and what we might call *mere generalisation* over particular instances of the phenomenon. Very roughly speaking, in the former case a generalisation is formed by referring to an explanatorily potent property that explains various instances of the phenomenon; in the latter, some feature that is present in each case, but does *not* explain the phenomenon, is exploited to formulate a generalisation. It is the deflationist’s contention that the truth predicate only plays the latter, merely generalising, role in the claim that true belief facilitates success.

Mere generalisation is an odd tool. Most of the time, if we generalise over particular instances of a phenomenon by picking up on an explanatorily *irrelevant* feature, what we’ll end up with is just a bad generalisation. I might, for example, generalise over Alison’s and Callum’s practical success by exploiting the fact that both have 6-letter names: ‘having a 6-letter name facilitates practical success’, I might say. This is a terrible generalisation; and it is so precisely *because* the feature so highlighted is explanatorily impotent when it comes to practical success. Good generalisations are typically a sign that the property in question *is* explanatorily relevant; and the truth generalisation is a good one.

There are, however, cases where good—or, at least, better—generalisations can be formulated using explanatorily irrelevant features, since there can be *correlation* without explanation. One way in which this is possible is if the correlation is merely coincidental. For example, a disproportionate number of recent U.S. presidents have been left-handed. We might formulate a generalisation thus: ‘left-handedness facilitates electoral success in the U.S.’; and insofar as we are able to read this as a *mere* generalisation, it might be true enough.^14^ But mere generalisation in this case is only permissible because there is no explanation forthcoming, i.e. because the correlation is coincidental. Presumably, then, the deflationist will want to deny the analogy between being left-handed and electoral success on the one hand, and true belief and practical success on the other; otherwise the correlation becomes brute and inexplicable because coincidental. There are many coincidental correlations in this world, but this is not one of them.

However, the only kind of case I can think of in which good generalisations can be formulated without explanation, but where the relevant correlation is nonetheless plausibly non-coincidental, is when there is a third-factor explanation. For example, vehicles with more safety equipment might be involved in proportionately fewer fatal collisions; but not because the safety equipment is effective, but because the type of person safety-conscious enough to purchase this kind of car is also more likely to drive carefully. We can offer the generalisation: ‘cars with more safety equipment are involved in fewer fatal collisions’; and this might be a good (if potentially misleading) generalisation despite the lack of explanation. What *explains* the fewer collisions is the safety-conscious drivers; and since this also *explains* why they own this type of car, it plausibly explains the correlation.^15^ As far as I can tell, then, if the deflationist is to maintain that truth does not explain practical success despite maintaining that this generalisation picks out a genuine and non-coincidental correlation, she must be committed to there being some such third-factor explanation.

This suggestion is quite implausible. We are to believe that there is some third factor, X, which explains why rational agents typically perform successful actions (presumably within certain parameters); and *also* explains why rational agents tend to believe the truth (presumably within those same parameters).^16^ That is, we are to believe that we *could explain* the practical success of actions performed by rational agents entirely in terms of X; but instead (presumably for expressive ease) offer a generalisation formulated in terms of the merely correlated property of truth. Given belief’s role in the explanation of rational behaviour, the explanation of practical success in terms of X must go via the agents’ beliefs: X will explain why agents tend to have success-conducive beliefs within the relevant parameters. But despite the fact that X *also* explains why these very beliefs tend to be true, the explanation of the agent’s success will *not* mention the truth of said beliefs. The truth of the beliefs thus becomes bizarrely epiphenomenal. And we might reasonably ask why it is that X, which produces success-conducive beliefs, also happens to produce true beliefs. Is it a coincidence? Why does it not produce success-conducive false beliefs? Are there none? Why not?

Either way, the main problem with this proposal is that it’s simply implausible that there is any such X that can, alone (i.e. without reference to truth), explain why the agent tends to be successful. As mentioned, this explanation will go via the success-conducive beliefs, but not their truth (which is epiphenomenal). It must, rather, go via their *causal profile*; the beliefs will have the same causal profile whether or not they are true, after all. But the causal profile of the beliefs X produces can only plausibly explain *why the agent performs the actions she performs*; and, as emphasised, this is merely the first step in the explanation; it is not to explain *why the actions she performs are successful*. Explaining the *success* of the actions thus performed requires explaining why the actions tend to go on to satisfy her desires. There is nothing in the causal profile of the beliefs *alone* that explains this: it requires the contribution of the world.

Wrenn (2011: 468–469) is surely right when he points out that evolution will select against those creatures whose beliefs tend to lead to unsuccessful actions. Presumably, then, evolution might select for some X that produces success-conducive beliefs; and this might “explain” why X produces success-conducive beliefs. But this evolutionary “explanation” is phenotypical, not genotypical: we are told what feature the beliefs and production mechanisms are selected for (producing successful action), but not what property they possess that gives rise to this feature. Why is it *these* beliefs, selected for by *this* mechanism, that lead to successful action? (Granted a cheetah’s legs are selected for running fast; but what is it about *these* legs that enable it to run fast?)^17^ The obvious explanation, given the correlation with true beliefs, is that X is selected for producing *true* beliefs precisely because *true* beliefs are useful for bringing about practical success. It is not merely the beliefs’ causal profile that explains the practical success; but the beliefs standing in the right relation to the world. But then X is not a third-factor after all: X explains why the agents tend to have true beliefs, which in turn explains why the agents tend to be successful. This picture is far more appealing, and once again it is only antecedent deflationary commitments that could push us to resist it.

There are other reasons to be sceptical of such third-factor proposals. For example, it is not clear that any third-factor explanation could have the robust modal profile that the truth generalisation possesses. Indeed, it’s not even obvious that correlations with third-factor explanations are truly non-coincidental, or non-coincidental in the right way. But in any case, it is not clear how a third-factor explanation could hope to solve the central problem of the paper: the Alison/Callum problem. We can stipulate that Alison and Callum are physically and psychologically identical, so if X explains why Alison has the (success-conducive) beliefs she has, then it explains why Callum has the (success-conducive) beliefs he has. Assuming that we are within the usual parameters, Alison’s beliefs are true as usual; while, unusually, Callum’s belief are false. But this is of no explanatory significance, as far as the success is concerned: X *alone* explains the practical success of any action (as well as, incidentally, the truth of Alison’s beliefs). So, if X explains Alison’s success, then it explains Callum’s; and if it doesn’t explain Callum’s, it doesn’t explain Alison’s. In this way, a third-factor explanation cannot hope to break the explanatory tie between the coincidental and non-coincidental cases presented.

## Conclusion

Maintaining that the sole role of truth in the claim that true belief facilitates practical success is as a device for generalisation requires showing that individual instances of practical success can be explained without invoking truth (or, at least, anything more than a relevant instance of the schema). The deflationist has traditionally offered seductive individualised explanations that seem to leave no explanatory work for truth. I have argued that these individualised explanations are problematically incomplete, as shown by the fact that we can give such individualised explanations when the practical success is merely coincidental. The contrast between a non-coincidence and a coincidence is an explanatory one; and I have argued that the deflationist’s refusal to explain the non-coincidental instances of practical success in terms of the truth of the agents’ beliefs renders her incapable of establishing an explanatory contrast between the cases. The inflationist, meanwhile, is entitled to the obvious explanatory resources required to recover the distinction. Pending some future deflationary response, then, the deflationary construal of the principle that true belief facilitates practical success is to be rejected; and with it the deflationary conception of truth. This is a major dialectical victory for inflationism.

I think the deeper lesson here is that the deflationist’s response strategy manifests an underlying confusion with regards to *explanation*. As we have seen, it is patently not enough to explain a *non*-*coincidental* phenomenon to explain each individual instance of it and then generalise over these explanations by highlighting some feature that they share in common, *but which is explanatorily irrelevant*. *Mere generalisation* is only appropriate for cases of *mere correlation* of one form or another. I’m not sure it has been appreciated that endorsing this “merely generalising” view hereby brings with it substantial—and, I have suggested, quite implausible—commitments as to the explanatory status *of the correlation* between true belief and practical success. So while my conclusion officially leaves the inflationist with the upper-hand and the deflationist with work to be done, my suspicion—with which I hope the reader is now sympathetic—is that the deflationary project is better off abandoned.

## Footnotes

1 Properly speaking, ‘deflationism’ picks out a family of theories. For ease of presentation, I address the argument here towards the most prominent varieties: disquotationalism and minimalism. Whatever their differences, deflationists are characteristically, if not constitutively, united in their rejection of the explanatory potency of truth.

2 The distinction between psychological and effective pertinence is due to Kitcher (2002: 358–359) and the terminology is Wrenn’s (2011: 460). My own definitions elaborate substantially on their sources; there are subtle variations between the accounts, but they need not delay us here.

3 In fact, psychological pertinence as it stands is too simple to play this role in full generality, being defined in terms of binary beliefs. We can straightforwardly define a more sophisticated notion of psychological pertinence in terms of credence that is apt to play the role more widely:
*Psychological pertinence**
An action A_i_ is psychologically pertinent* to an outcome O for a rational agent S in a context C iff: (i) A_i_ ∈ {A_1_, …, A_n_}, where A_1_, …, A_n_ are S’s options for action in C; and (ii) for any j ≠ i, where A_j_ ∈ {A_1_, …, A_n_}, the credence that S assigns to S’s A_j_-ing resulting in O is less than or equal to the credence S assigns to S’s A_i_-ing resulting in O. While a more adequate rationalisation condition, talking in terms of psychological pertinence* (and the relevantly altered notion of effective pertinence) results in unnecessary complication. All that we require are the simple cases in which (non-starred) psychological pertinence is satisfactory.

4 Compare Wrenn (2011: 454): ‘*success* occurs when a particular action has its intended outcome’.

5 Note that the success of A with respect to D is only sufficient for the coincidence of psychological and effective pertinence, not necessary.

6 Damnjanovic describes the following as the deflationist’s ‘standard response’; see Damnjanovic (2005) and the citations therein.

7 If there can be a deflationary theory of truth that takes beliefs as the primary truth bearers, then this will straightforwardly be given by the relevant instance of the deflationary schema.

8 We are granting the deflationist the view that such beliefs appear in the intentional explanations of rational actions; Wrenn (2011) contends that this “single action-guiding belief” view of intentional explanation is controversial.

9 The *style* of this response to the Success Argument exemplifies a powerful dialectical move in any dispute between inflationists and deflationists. For any purported explanatory or normative role for truth, the deflationist seeks to eliminate mention of truth from any particular instance of the explanation or norm in favour of the relevant instance of their preferred schema; and thereby maintain that the role of the truth predicate is merely as an expressive device for indirect reference to and compendious generalisation over such instances. Much of Horwich’s (1998) is occupied with applying precisely this strategy to various inflationary arguments. Another particularly neat example is Dodd’s (1999) response to Wright’s (1992) argument that truth marks a distinctive norm of assertion: ‘[‘One should assert only what is true’] is just shorthand for an infinite conjunction of claims of the form… [‘]One should assert that snow is white only if snow is white; one should assert that coal is black only if coal is black, etc.[’]’ (1999: 296).

10 A recent and very insightful discussion of coincidence starts with the sentence, ‘It is a truism that coincidences cannot be explained’—see Lando (2016) and the citations therein. Lando suggests that this truism ought to be finessed, because there are coincidences with a common cause; in particular, she suggests coincidences may be cases where there is a salient *relational property* between the instances that cannot be explained (e.g. that the Sun and Moon appear *the same* size; that two people look *alike*; and so on). I like this suggestion, and it accords well with what I say below (in Callum’s case, it is a coincidence that the psychologically and effectively pertinent action are *the same*); but I need not rely on it.

11 Whether or not conjunctive quasi-explanations are really explanations, moving forward I’ll largely use ‘explanation’ to mean the kind of explanation proper that is appropriate for non-coincidences in particular.

12 It is, of course, controversial exactly what an explanation proper consists in. We can remain neutral on such issues.

13 Note that (1A)–(9A) may still be a *proper part* of the explanation of Alison’s success; as indicated above, there need not be anything strictly speaking *incorrect* about a conjunctive quasi-explanation when the explanandum is non-coincidental. It is rather that it is problematically *incomplete* (in the sense that there are explanatorily premises missing), in such a way that what we have is not really an *explanation* of the non-coincidence until it is supplemented.

14 To this end, ‘facilitation’ is probably the wrong word, but given that the deflationist typically takes herself to be entitled to it in the truth case, it is unclear why we should not be entitled to it here. This, I think, just highlights how odd the deflationist’s suggestion is; and how this oddness is often masked by the language used.

15 I am granting this for the sake of argument; we ought to note the structural similarity here with the conjunctive quasi-explanations of one-off coincidences.

16 X might most plausibly be a psychological (especially a belief-forming) mechanism. Wrenn (2011: 468) seems to have something like this in mind.

17 There are parallels—here and throughout—with the dispute in the scientific anti-/realism literature as to whether or not we need postulate the truth of our best theories to explain their success. The critical difference is that here the truth of the relevant representations, the beliefs, is not in doubt. My present concern is to press the implausibility of *simultaneously* conceding the truth of the success-conducive beliefs we have while *denying* the explanatory relevance of said truth to that success.

## References

[CR1] Damnjanovic N (2005). Deflationism and the success argument. Philosophical Quarterly.

[CR2] Dodd J (1999). There is no norm of truth: A minimalist reply to Wright. Analysis.

[CR3] Field H (1972). Tarski’s theory of truth. Journal of Philosophy.

[CR4] Horwich P (1998). Truth.

[CR5] Kitcher P (2002). On the explanatory role of correspondence truth. Philosophy and Phenomenological Research.

[CR6] Lando T (2016). Coincidence and common cause. Noûs.

[CR7] Wrenn C (2011). Practical success and the nature of truth. Synthese.

[CR8] Wright C (1992). *Truth and objectivity*.

